# Hemodialysis Central Venous Catheter-Associated Bloodstream Infection Caused by *Stenotrophomonas maltophilia* Treated with Cefiderocol and Levofloxacin After Failure of Trimethoprim–Sulfamethoxazole Monotherapy and Device Replacement

**DOI:** 10.3390/antibiotics15030265

**Published:** 2026-03-04

**Authors:** Simone Meini, Alberto Antonelli, Benedetta Longo, Maddalena Mura, Elisabetta Andreoli, Angeliki Kanaki, Giulia Grassi, Claudia Niccolai, Bruno Viaggi, Gian Maria Rossolini

**Affiliations:** 1 Internal Medicine Unit, Felice Lotti Hospital of Pontedera, Azienda Unità Sanitaria Locale Toscana Nord-Ovest, 56121 Pisa, Italy; benedetta.longo@uslnordovest.toscana.it (B.L.); maddalena.mura@uslnordovest.toscana.it (M.M.); 2 Microbiology and Virology Unit, Florence Careggi University Hospital, 50134 Florence, Italy; alberto.antonelli@unifi.it (A.A.); claudia.niccolai@unifi.it (C.N.); gianmaria.rossolini@unifi.it (G.M.R.); 3 Department of Experimental and Clinical Medicine, University of Florence, 50134 Florence, Italy; 4 Microbiology Laboratory, Felice Lotti Hospital of Pontedera, Azienda Unità Sanitaria Locale Toscana Nord-Ovest, 56121 Pisa, Italy; elisabetta.andreoli@uslnordovest.toscana.it; 5 Nefrology Unit, Felice Lotti Hospital of Pontedera, Azienda Unità Sanitaria Locale Toscana Nord-Ovest, 56121 Pisa, Italy; angeliki.kanaki@uslnordovest.toscana.it (A.K.); giulia.grassi@uslnordovest.toscana.it (G.G.); 6 ICU Department, Careggi Hospital, 50134 Florence, Italy; viaggib@aou-careggi.toscana.it

**Keywords:** *Stenotrophomonas maltophilia*, bloodstream infection, cefiderocol, levofloxacin, trimethoprim–sulfamethoxazole, checkerboard, synergism

## Abstract

**Background**: *Stenotrophomonas maltophilia* infections represent a clinical challenge in treating frail and immunocompromised patients. Alternatives to trimethoprim–sulfamethoxazole (SXT) are needed, with cefiderocol (FDC) representing a promising option, but clinical evidence is limited; moreover, data to support the superiority of mono or combination therapy are lacking. **Case presentation**: We describe the case of a 55-year-old female patient with a tunneled hemodialysis central venous catheter (HD-CVC)-associated bloodstream infection caused by *S. maltophilia* that, after failure of a prolonged SXT monotherapy, was successfully treated by HD-CVC replacement followed by intravenous cefiderocol (FDC) and levofloxacin (LVX). **Conclusions:** FDC represents an interesting option for complex cases of *S. maltophilia* bloodstream infections, and the combination with LVX might add benefit in cases associated with biofilm formation on intravascular devices.

## 1. Introduction

*Stenotrophomonas maltophilia* is an aerobic, glucose-non-fermenting, Gram-negative bacillus, ubiquitous in the environment. *S. maltophilia* is able to survive on humid surfaces and shows a great propensity for growing as biofilm on biotic and abiotic surfaces (e.g., catheters, endoscopes, percutaneous drainages, hemodialysis and ventilation circuits), conferring protection from host defenses and antimicrobial treatment [1,2]. *S. maltophilia* often represents a colonizing organism; thus, colonization must be carefully distinguished from infection to avoid unnecessary antibiotic use.

Despite being generally considered a low-virulence pathogen, the crude mortality rate in cases of bloodstream infection (BSI) ranges from 14 to 69% [3,4,5]; moreover, it can cause hemorrhagic pneumonia in patients with hematologic malignancies [6]. The characteristics of infected patients, often immunocompromised or affected by chronic lung conditions such as cystic fibrosis or by end-stage renal disease requiring hemodialysis, play a crucial role in explaining negative clinical outcomes.

*S. maltophilia* displays intrinsic resistance to several antibiotics due to the presence of an impressive number of resident antimicrobial resistance mechanisms [7]: two inducible chromosomal genes coding the L1 metallo-β-lactamase that hydrolyzes penicillins, cephalosporins, and carbapenems, but not aztreonam, and the L2 serine β-lactamase that hydrolyzes extended-spectrum cephalosporins and aztreonam and can be inhibited by avibactam [2]; chromosomal acetyl-transferase enzymes causing intrinsic resistance to aminoglycosides; multidrug efflux pumps that can reduce the activity of trimethoprim–sulfamethoxazole (SXT), tetracyclines, and fluoroquinolones; and chromosomal Smqnr genes that reduce the activity of fluoroquinolones [2,8,9,10,11]. Moreover, acquired resistance genes may also be present, conferring resistance to many agents, including SXT [12,13].

A “standard of care” for *S. maltophilia* infections is currently not available [14]. EUCAST provides clinical breakpoints only for SXT (S ≤ 0.001, R > 2 mg/L) due to insufficient evidence for other antimicrobials [15], while CLSI has established breakpoints for five additional agents, including cefiderocol (FDC), chloramphenicol, levofloxacin (LVX), minocycline, and ticarcillin-clavulanate. Levofloxacin has been proposed as a useful alternative option to SXT for the treatment of bloodstream and lower respiratory tract infections [16,17].

Recently, the view that SXT should be considered the agent of choice has been challenged in a comprehensive review [12]. EUCAST found FDC to be a potential alternative, but the clinical evidence is still too limited to allow the definition of a breakpoint [12]; moreover, sufficient data to support the superiority of mono or combination therapies are lacking.

Due to the limited supportive data for any individual agent and pending robust evidence, the most recent IDSA guidance [6] suggests the use of two agents among FDC, minocycline, SXT, or LVX. Another option is represented by the combination of ceftazidime–avibactam and aztreonam, or, rationally, by the newly available combination aztreonam–avibactam.

The *in vitro* activity of FDC against *S. maltophilia* is comparable to the activity against Enterobacterales [15]; moreover, PK/PD data and animal models are encouraging [6]. Testing FDC is challenging because broth microdilution MIC determination must be performed in iron-depleted Mueller–Hinton broth. As reported by EUCAST, isolates with MIC values ≤ 0.5 mg/L are mostly devoid of resistance mechanisms, while isolates with MIC values 1–2 mg/L have acquired resistance mechanisms, which may result in impaired clinical response, and isolates with MIC values > 2 mg/L will likely be resistant.

To date, since data on the use of FDC as monotherapy or in combination therapy are lacking, any clinical data supported by adequate microbiological susceptibility tests are crucial to help fill the gap in the evidence regarding the most effective strategy.

Here, we report on a hemodialysis patient with a persistent bloodstream infection caused by *S. maltophilia*, not responding to prolonged treatment with SXT, and successfully treated by adding a combination of FDC and LVX and removing her tunneled hemodialysis central venous catheter (HD-CVC). In vitro microbiological analyses on the synergism of this combination are also provided.

## 2. Results

### 2.1. Case Description

A 55-year-old female was referred on 6 January 2023 by the local dialysis center to the emergency department of the Hospital of Pontedera (Tuscany, Italy) due to the persistence for more than one month of fever with chills after each dialytic session. Several blood cultures had returned positive for *S. maltophilia* during this period (on 28 and 30 November, 9 and 26 December 2022, and 2 January 2023). A prolonged treatment with SXT, at the daily dosage of 240 mg of trimethoprim (TMP) component intravenously after dialysis, and of 320 mg orally on other days, had been administered during this period with no resolution. The patient suffered from hypertension and critical limb ischemia of the lower limbs; because of renal amyloidosis with end-stage renal disease, she was initially treated with peritoneal dialysis from January 2020 to September 2021. Subsequently, due to recurrent peritonitis, she underwent three-weekly hemodialysis through a tunneled catheter (HD-CVC) inserted in the right subclavian vein.

At admission to the hospital, trans-thoracic echocardiography showed a moderate insufficiency on the aortic valve and a suspected vegetation on the right coronary cusp, but a subsequent trans-esophageal echocardiography excluded endocarditis. Blood tests showed the absence of leukocytosis, but C-reactive protein and procalcitonin were significantly increased (74.7 mg/L and 73.4 ng/mL, respectively). Monotherapy with intravenous SXT, at an increased dosage of 480 mg (of TMP component) per day in three divided doses (calculated for a weight of 54 kg), was initially provided for three days, until removal of the subclavian HD-CVC, performed on 9 January 2023, while a temporary femoral venous catheter was simultaneously inserted to provide access for dialysis and antibiotic treatment. The culture of the removed device grew *S. maltophilia* (isolate OFL-Po-01-23). On 10 January, the patient was stable from the hemodynamic and the respiratory point of view; the inflammatory indices were slightly decreasing, but considering that the isolate from the blood culture performed on 2 January had in the meantime been reported resistant to SXT (MIC 8/152 mg/L), and pending the results of cultures from the HD-CVC removed the day before, we decided to administer a combination of intravenous levofloxacin 500 mg q48h and cefiderocol 1 g q12h.

On 15 January, the values of C-reactive protein and procalcitonin significantly dropped to 15.3 mg/L and 4.38 ng/mL, respectively. On 16 January, the patient decided to leave the hospital and was discharged with instructions to continue oral SXT 160/800 mg q12h and oral LVX 500 mg q48h for 10 days. The choice to continue SXT in combination with LVX was based on the susceptibility to the two drugs exhibited by the *S. maltophilia* isolated from the HD-CVC and on the possibility of oral treatment, while presuming that the anti-biofilm activity of LVX [18] might contribute to preventing device reinfection.

On 27 January, a control visit found the patient in good clinical condition; fever had no longer been reported, the level of procalcitonin was 0.7 ng/mL, and oral antibiotic therapy with LVX and SXT was stopped after a 10-day course.

A control blood culture, taken on 15 March 2023, was negative. At several follow-up visits, the patient was found in good condition, and an arteriovenous fistula was eventually inserted.

At the time of writing this case report, the patient had not reported any further infections caused by *S. maltophilia*.

### 2.2. Microbiological Analyses

First-level microbiological analyses had been performed at the local microbiology laboratory. Species identification was performed with MALDI-ToF mass spectrometry (Bruker, Billerica, MA, USA), and antimicrobial susceptibility testing (AST) was performed with a semi-automated system (Vitek2, bioMérieux, Marcy-L’Etoile, France).

The *S. maltophilia* isolated from the HD-CVC on 9 January 2023 (isolate OFL-Po-01-23) was sent to a regional reference laboratory of clinical microbiology (Careggi University Hospital, Florence, Italy) for further characterization. AST was performed by broth microdilution using cation-adjusted Mueller–Hinton broth and a lyophilized panel (MERLIN Diagnostika GmbH, Bornheim, Germany), except for FDC, which was tested using an iron-depleted medium as previously described [19]. AST results were interpreted according to the EUCAST clinical breakpoints (v 13.0, 2023, those available at the time), except for FDC and LVX, which were interpreted according to CLSI (M100 Ed 33). A checkerboard assay to assess the potential synergism of LVX and FDC was performed as previously described [20] using iron-depleted Mueller–Hinton broth. The concentration ranges of LVX and FDC were 0.125–8 mg/L and 0.015–16 mg/L, respectively. Results were interpreted based on the Fractional Inhibitory Concentration Index as follows: FICI ≤ 0.5, synergy; FICI 0.5–4.0, no interaction; FICI > 4.0, antagonism [20,21,22].

AST results reported by the local laboratory on *S. maltophilia* isolates from blood cultures taken on 28 and 30 November and on 9 December 2022 showed SXT MICs ≤ 1/19 mg/L, while isolates from blood cultures taken on 26 December 2022 and 2 January 2023 showed SXT MICs of 4/76 mg/L and 8/152 mg/L, respectively.

*S. maltophilia* OFL-Po-01-23, isolated from the HD-CVC removed on 9 January 2023 and tested at the regional reference laboratory, was susceptible to SXT according to EUCAST (MIC ≤ 1/19 mg/L), and to FDC and LVX according to CLSI (MICs 0.25 mg/L for both drugs).

The cefiderocol/levofloxacin combination showed a FICI value of 0.75, revealing neither synergistic nor antagonistic interaction.

## 3. Discussion

This case presents several clinically challenging issues that can be related to infections caused by *S. maltophilia*, which are burdened by high morbidity and mortality in frail patients predisposed to these infections, whereby studies of adequate robustness addressing the best “standard of care” are lacking.

SXT represents the agent with the best-documented clinical activity and has been historically the treatment of choice for infections caused by *S. maltophilia*, but not all patients can be treated, commonly due to sulphonamide intolerance or allergy. Moreover, the dosages of SXT are difficult to optimize in patients affected by chronic kidney disease and needing hemodialysis. TMP competes for tubular secretion of creatinine, with a secondary increase in the level of about 30% [23], blocks distal tubular secretion of potassium [24], with risk of hyperkalemia, and induces hyponatremia. To date, the Sanford Guide for Antimicrobial Therapy does not recommend SXT use in hemodialysis patients and suggests that, if used, a dose of 5–10 mg/kg (of TMP component) per day be administered.

Studies carried out *in vitro* in a chemostat model showed that SXT monotherapy, even at higher doses, achieved limited activity against susceptible *S. maltophilia* [25]. Therefore, identification of new treatment strategies is urgently needed, mainly for frail and multimorbid patients.

In our case, before hospitalization, the patient was treated with SXT with a mean of 5.3 mg/kg of TMP component per day and subsequently, during hospitalization, was treated intravenously with 8.9 mg/kg of TMP component per day in three divided doses. The treatment was well tolerated, without adverse events, but we observed a progressive increase in the MICs of SXT from the blood cultures collected in the meantime. We can hypothesize that, under the continuous selective pressure of SXT, a subpopulation of resistant mutants was selected (and isolated from the blood), while most of the bacteria present in the biofilm remained susceptible. Taking into account the possible further selection of resistant subpopulations to SXT, and in accordance with the current IDSA guidance on combination therapy [6], we decided to start an association between FDC and LVX. In fact, at the time the patient was treated, IDSA suggested for moderate–severe infections LVX (the only fluoroquinolone with established CLSI clinical breakpoints) in combination with a second active agent (SXT, minocycline, tigecycline, FDC) [26].

Fluoroquinolones show anti-biofilm activity against *S. maltophilia* higher than that of SXT and therefore could represent useful agents for combination regimens in certain clinical scenarios, such as catheter-related bacteremia [16,18]. In a large Italian surveillance program conducted from 2014 to 2018, 100% of the 127 *S. maltophilia* isolates were susceptible to FDC [8].

Surely, the possibility of prompt removal of an infected central venous catheter is crucial for effective infection source control, but in real life it is not always possible to do it immediately; moreover, usually there is the need to insert another device equally at risk of infection relapse, and effective bactericidal antibiotic therapy is advisable. In our case, removing the infected tunneled HD-CVC as soon as possible likely played a crucial role in resolving the infection, but the administration of FDC plus LVX may also have played a role in preventing device reinfection by eradicating possible residual bacteria in the bloodstream. Moreover, LVX might have had a further role in protecting the new central venous catheter from reinfection because of its anti-biofilm activity [16,18].

*In vitro* data from a checkerboard assay showed a FICI value of 0.75; this value, which has been considered in the past as “partial synergism” [22,27], should be considered as indicative of neither synergistic nor antagonistic interaction (FICI 0.5–4.0) [20,21].

The main limitations of this work are represented by the fact that it refers to a single case observation and that only a single isolate had been preserved for further microbiological analysis.

## 4. Conclusions

This case suggests that cefiderocol in combination with levofloxacin can represent an interesting option for the treatment of complex cases of *S. maltophilia* infections. Further data will be needed to show which antibiotic combinations are most effective.

## Data Availability

The original contributions presented in this study are included in the article. Further inquiries can be directed to the corresponding author.

## Abbreviations

The following abbreviations are used in this manuscript:

AST	Antimicrobial Susceptibility Testing
CLSI	Clinical & Laboratory Standards Institute
EUCAST	European Committee on Antimicrobial Susceptibility Testing
FDC	Cefiderocol
FICI	Fractional Inhibitory Concentration Index
HD-CVC	Hemodialysis central venous catheter
LVX	Levofloxacin
PK/PD	Pharmacokinetics/pharmacodynamics
SXT	Trimethoprim–sulfamethoxazole
TMP	Trimethoprim

## References

[1] de Oliveira-Garcia D., Dall’Agnol M., Rosales M., Azzuz A.C.G.S., Alcántara N., Martinez M.B., Girón J.A. (2003). Fimbriae and adherence of *Stenotrophomonas maltophilia* to epithelial cells and to abiotic surfaces. Cell. Microbiol..

[2] Brooke J.S. (2012). *Stenotrophomonas maltophilia*: An emerging opportunistic pathogen. Clin. Microbiol. Rev..

[3] Brooke J.S. (2021). Adevances in the microbiology of *Stenotrophomonas maltophilia*. Clin. Microbiol. Rev..

[4] Falagas M.E., Kastoris A.C., Vouloumanou E.K., Rafailidis P.I., Kapaskelis A.M., Dimopoulos G. (2009). Attributable mortality of *Stenotrophomonas maltophilia* infections a systematic review of the literature. Future Microbiol..

[5] Mojica M.F., Rutter J.D., Taracila M., Abriata L.A., Fouts D.E., Papp-Wallace K.M., Walsh T.J., LiPuma J.J., Vila A.J., Bonomo R.A. (2019). Population structure, molecular epidemiology, and β-lactamase diversity among *Stenotrophomonas maltophilia* isolates in the United States. mBio.

[6] Tamma P.D., Heil E.L., Justo J.A., Mathers A.J., Satlin M.J., Bonomo R.A. (2024). Infectious Diseases Society of America 2024 Guidance on the Treatment of Antimicrobial-Resistant Gram-Negative Infections. Clin. Infect. Dis..

[7] Crossman L.C., Gould V.C., Dow J.M., Vernikos G.S., Okazaki A., Sebaihia M., Saunders D., Arrowsmith C., Carver T., Peters N. (2008). The complete genome, comparative and functional analysis of *Stenotrophomonas maltophilia* reveals an organism heavily shielded by drug resistance determinants. Genome Biol..

[8] Stracquadanio S., Torti E., Longshaw C., Henriksen A.S., Stefani S. (2021). *In vitro* activity of cefiderocol and comparators against isolates of Gram-negative pathogens from a range of infection sources: SIDERO-WT-2014–2018 studies in Italy. J. Glob. Antimicrob. Resist..

[9] Okazaki A., Avison M.B. (2007). Aph(3′)-IIc, an aminoglycoside resistance determinant from *Stenotrophomonas maltophilia*. Antimicrob. Agents Chemother..

[10] Gordon N.C., Wareham D.W. (2010). Novel variants of the *Smqnr* family of quinolone resistance genes in clinical isolates of *Stenotrophomonas maltophilia*. J. Antimicrob. Chemother..

[11] Alonso A., MartínEz J.L. (2000). Cloning and characterization of SmeDEF, a novel multidrug efflux pump from *Stenotrophomonas maltophilia*. Antimicrob. Agents Chemother..

[12] Turnidge J., Gatermann S., Kahlmeter G., Cantón R., Wootton M., Giske C.G. (2025). on behalf of the EUCAST. Rationale for contemporary antimicrobial treatment of *Stenotrophomonas maltophilia*: A narrative review. CMI Commun..

[13] Boncompagni S.R., Riccobono E., Cusi M.G., Di Pilato V., Rossolini G.M. (2025). Evidence of dissemination of a clc-type integrative and conjugative element to *Stenotrophomonas maltophilia*, mediating acquisition of *sul1* and other resistance determinants. Antimicrob. Agents Chemother..

[14] Falagas M.E., Valkimadi P.E., Huang Y.T., Matthaiou D.K., Hsueh P.R. (2008). Therapeutic options for *Stenotrophomonas maltophilia* infections beyond co-trimoxazole: A systematic review. J. Antimicrob. Chemother..

[15] https://www.eucast.org/fileadmin/eucast/pdf/breakpoints/v_15.0_Breakpoint_Tables.pdf.

[16] Cho S.Y., Kang C.-I., Kim J., Ha Y.E., Chung D.R., Lee N.Y., Peck K.R., Song J.-H. (2014). Can levofloxacin be a useful alternative to trimethoprim-sulfamethoxazole for treating *Stenotrophomonas maltophilia* bacteremia?. Antimicrob. Agents Chemother..

[17] Sarzynski S.H., Warner S., Sun J., Matsouaka R., Dekker J.P., Babiker A., Li W., Lai Y.L., Danner R.L., Fowler V.G. (2022). Trimethoprim-Sulfamethoxazole Versus Levofloxacin for *Stenotrophomonas maltophilia* Infections: A Retrospective Comparative Effectiveness Study of Electronic Health Records from 154 US Hospitals. Open Forum Infect. Dis..

[18] Santos Carvalhais B.E., Souza E., Silva C., Dos Santos K.V. (2021). Effect of antimicrobials on *Stenotrophomonas maltophilia* biofilm. Future Microbiol..

[19] Hackel M.A., Tsuji M., Yamano Y., Echols R., Karlowsky J.A., Sahm D.F. (2019). Reproducibility of broth microdilution MICs for the novel siderophore cephalosporin, cefiderocol, determined using iron-depleted cation-adjusted Mueller-Hinton broth. Diagn. Microbiol. Infect. Dis..

[20] Boncompagni S.R., Micieli M., Di Maggio T., Aiezza N., Antonelli A., Giani T., Padoani G., Vailati S., Pallecchi L., Rossolini G.M. (2022). Activity of fosfomycin/colistin combinations against planktonic and biofilm Gram-negative pathogens. J. Antimicrob. Chemother..

[21] Gómara M., Ramón-García S. (2019). The FICI paradigm: Correcting flaws in antimicrobial *in vitro* synergy screens at their inception. Biochem. Pharmacol..

[22] Hall M., Middleton R.F., Westmacott D. (1983). The fractional inhibitory concentration (FIC) index as a measure of synergy. J. Antimicrob. Chemother..

[23] Nakada T., Kudo T., Kume T., Kusuhara H., Ito K. (2018). Quantitative analysis of elevation of serum creatinine via renal transporter inhibition by trimethoprim in healthy subjects using physiologically-based pharmacokinetic model. Drug Metab. Pharmacokinet..

[24] Alappan R., Buller G.K., Perazella M.A. (1999). Trimethoprim-sulfamethoxazole therapy in outpatients: Is hyperkalemia a significant problem?. Am. J. Nephrol..

[25] Lasko M.J., Tabor-Rennie J.L., Nicolau D.P., Kuti J.L. (2022). Trimethoprim/sulfamethoxazole pharmacodynamics against *Stenotrophomonas maltophilia* in the in vitro chemostat model. J. Antimicrob. Chemother..

[26] Tamma P.D., Aitken S.L., Bonomo R.A., Mathers A.J., Van Duin D., Clancy C.J. (2022). Infectious Diseases Society of America Guidance on the Treatment of AmpC β-Lactamase-producing Enterobacterales, Carbapemen-Resistant *Acinetobacter baumaannii*, and *Stenotrophomonas maltophilia* Infections. Clin. Infect. Dis..

[27] Di X., Wang R., Liu B., Zhang X., Ni W., Wang J., Liang B., Cai Y., Liu Y. (2015). *In vitro* activity of fosfomycin in combination with colistin against clinical isolates of carbapenem-resistant *Pseudomas aeruginosa*. J. Antibiot..

